# Preoperative peripheral plasma fibrinogen level is an independent prognostic marker in penile cancer

**DOI:** 10.18632/oncotarget.12563

**Published:** 2016-10-11

**Authors:** Chengquan Ma, Yaguang Zhou, Sufen Zhou, Kun Zhao, Bingxin Lu, Erlin Sun

**Affiliations:** ^1^ Department of Urology, The Second Hospital of Tianjin Medical University, Tianjin, China; ^2^ Department of Operation Room, The Second Hospital of Tianjin Medical University, Tianjin, China; ^3^ Department of Urology, Jixian Peoples Hospital, Tianjin, China; ^4^ Tianjin institute of Urology,The Second Hospital of Tianjin Medical University, Tianjin, China; ^5^ Department of Urology,Tianjin Nankai Hospital, Tianjin, China

**Keywords:** penile cancer, biomarker, fibrinogen, prognosis, survival

## Abstract

Background and aim: High levels of peripheral plasma fibrinogen have recently been revealed that related to poor clinical prognosis in various types of malignant tumors. The purpose of this research was to identify the prognostic significance of the preoperative peripheral serum fibrinogen level in patients with penile cell carcinoma.

Methods: This retrospective research included 72 penile cancer patients with date about their serum fibrinogen value before surgery who undergone either partial or radical penectomy at The 2nd Hospital of Tianjin Medical University between January 2002 to January 2012. They had a mean follow-up of 30.8 months. To determine the factors that were significant in predicting a patients prognosis, univariate and multivariate analyses were performed according to the Cox proportional hazards regression model.

Results: The 5-year cancer specific survival (CSS) rate was 62.4% of patients with preoperative fibrinogen levels below 340 mg/dl and 41.9% for those with higher levels (*p* = 0.001). Multivariate analysis revealed that the pathological T stage (*p* < 0.001), tumor grade (*p* = 0.036), postoperative chemotherapy (*p* = 0.041), nodal metastasis(*p* < 0.001), pathological type (*p* < 0.001) and fibrinogen (*p* = 0.023) were independent prognostic factors for survival. Patients with low fibrinogen level (<340mg/dl) had significantly longer CSS and the different survival rate were defined using the log-rank test.

Conclusions: The high preoperative peripheral serum fibrinogen level was related to poor survival in penile cancer patients. Fibrinogen may serve as a powerful predictor of CSS in penile cancer patients.

## INTRODUCTION

Penile cancer is a rare but ominous disease, accounting for 0 ~1% of all malignancies in men. Penile cancer is estimated 2,030 new cancer cases and 340 deaths in United States of 2016 [1] .It is relatively common in the Asia than Europe. According to poor hygiene and economic conditions, its incidence can reach 10% of all malignancies in Asian countries especially india as well as it is higher in Uganda [2]. The main prognostic factor of the penis is the extent of inguinal lymph node involvement [3]. There are no classical molecular biomarkers for clinical practice value of the penis until today. The soluble eptithelial antigen of penile tumor is short of sensitivity in the diagnosis of small size tumor and has poor prognostic significance for survival following operation [4]. The development of new tools for the prognostic is a challenge in uro-oncologic study, and it could provide benefits for tumor invasiveness and take positive measures to reduce harm from malignant

Lots of articles revealed that an interrelation exists between haemostatic factors and tumor biological behavior. The hypercoagulable state is considered to be correlative with malignancy that it has been showed a high proportion of 50% in patient with tumor. Fibrinogen, a 340-kDa glycoprotein that is synthesized in liver and is transformed into fibrin through the effect of activated thrombin, play an important role as a coagulation factor. An enormous number of procoagulant and fibrinolytic factors were discovered to be overexpressed in malignant tumors. Plasma fibrinogen as one member of these factors was involved in. Recent studies have revealed that elevated preoperative plasma fibrinogen levels are related to poor outcome in various types of malignancies; however, the clinical and prognostic value of preoperative plasma fibrinogen levels in patients with operable penile tumor has not yet been reported. The object of this retrospective research was to define the impact of fibrinogen levels at diagnosis on the prognosis of patient with penile cancer. To our knowledge we present the first comprehensive study of the prognostic role of preoperative fibrinogen for CSS.

## MATERIALS AND METHODS

### Patient and tumor date

This research included 72 patients with date about their fibrinogen value directly before partial or radical penectomy who underwent penile tumor surgery from January 2002 to January 2012 at The 2nd Hospital of Tianjin Medical University. The standard of histological tumor subtype was defined in the light of the 2010 UICC Classification. The histologic tumor grade is according to the classification by Broders. Patients who received perioperative chemoradiotherapy or radiotherapy were excluded. Our hospital and institute databases were used to obtain patient and tumor information, such as age, sex, TNM stage, regional lymph node involvement or distant metastasis, tumor histological subtype, fibrinogen value, tumor grade, and body mass index (BMI). Table 1 reveals the clinicopathological characteristics of the patients recruited in this study. Every included patient was followed up every 2-6 months thereafter to leave the hospital. All the patients signed informed consent about the use and publication of their date prior to operation. This research was affirmed by the ethics committee of the institution. All the blood samplings were got from less than 3 days in advance of operated patients. For assaying fibrinogen, we used the Clauss mode that is in view of the thrombin clotting time according to Datafai Fibrinogen and a CA7000 analyzer made in Japan (Sysmex Co-operation).

**Table 1:** Univariate and multivariate analysis for CSS

Factors	Univariate hazard ratio	95% CI	*p* value	Multivariate hazard ratio	95% CI	*p* value
Age (≥ 60 *vs*. < 60 years)	4.62	0.88-22.40	0.095			
BMI(≥ 24.3 *vs*. <24.3 kg/m^2) Tumour grade (G3–4 *vs*. G1–2)	1.75 2.84	0.66-14.25 1.17-31.36	0.22 0.006	1.54	1.075-26.440	0.036
Tumour stage (≥ pT2 *vs*. < pT2)	9.75	1.99-25.58	<0.001	4.46	1.22-15.36	<0.001
Postoperative Chemotherapy (No *vs*. Yes) Smoking≥5years (Yes *vs*. No) Nodal metastasis (N0 *vs*. N+) Pathological Type (squamous carcinoma *vs*. other carcinoma) Fibrinogen (≥ 340 *vs*. < 340 mg/dl)	2.08 1.74 11.70 13.32 6.97	1.52-64.83 1.22-16.66 4.96-49.91 6.606-21.432 1.84-22.97	0.032 0.029 <0.001 <0.001 <0.001	1.116 1.62 7.63 11.86 5.03	1.13-94.35 0.388-29.97 4.45-86.39 5.97-57.72- 1.74-62.30	0.041 0.943 <0.001 <0.001 0.023

### Statistical analysis

To evaluate the connection between the pre-therapeutic plasma fibrinogen level and clinicopathological factors, the Student's t-test was performed to acquire mean values with standard deviation (SD), whereas the Mann-Whitney U-test and chi-squared test were performed for categorized data and qualitative data, respectively. We selected 340 mg/dl as an optimal threshold value of fibrinogen according to a calculation using receiver operating characteristic curve analysis. To determine the factors that were significant in predicting a patient's prognosis, a method of multivariate analysis was performed in the light of the Cox proportional hazards regression model. And just the variables revealed to be statistically significant through univariate analysis (*p* < 0.05) were choose into the multivariate analysis. Kaplan-Meier survival curves were adopted and the different survival rates were defined using the log-rank test. The statistical analysis was performed by SPSS version 20.0 for Microsoft windows. All reported p values were 2-sided, and a *p* < 0.05 was considered to be statistically significant.

## RESULTS

Clinical characteristics of the 72 patients in this research are shown in Table 1. Median age was 67 years (range 37-86 years), and median follow-up was 30.8 months (range from 2-125 months). 27 patients (21.4%) died during the follow-up period. 64 (88.89%) of the tumors were squamous cell carcinoma, 8 (11.11%) of the tumors are other carcinomas. 30 (41.67%) of the tumors stage were pT≥2, 42 (58.33%) pT < 2. 57 (79.12%) of the tumors grade were G3/G4, 15(20.83%) G1/G2, 29 (40.28%) of the tumors were with nodal metastasis, 43(59.72%) without nodal metastasis. A total of 46 patients (63.89%) had smoking experience (>5years), and 26 (36.11%) of no-smoking or smoking less than 5 years.

**Figure 1:**
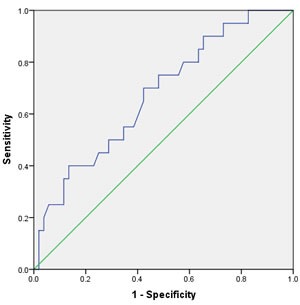
Receiver operating curve (ROC) for predicting patients’ prognosis with fibrinogen

**Figure 2:**
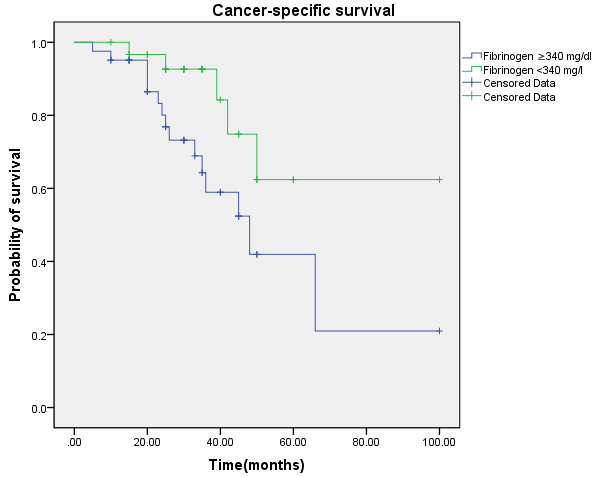
Cancer-specific survival (CSS) by plasma fibrinogen level between the ≥ 340 mg/dl and < 340 mg/dl fibrinogen groups

Univarate Cox regression analysis showed that - in contrast to advanced age (>60years; HR 4.62, 95% CI 0.88-22.4, *p* = 0.095), raising BMI (>24.3 kg/m2; HR 1.75, 95% CI 0.66-14.25, *p* = 0.22), high tumor stage(≥*p*T2; HR 9.75, 95% CI 1.99-25.58, *p* < 0.001), smoking≥5years (yes; HR 1.74, 95% CI 1.22-16.66, *p* = 0.029), pathological type (squamous carcinoma vs. other carcinoma, HR 13.32, 95% CI 6.606-21.432, < 0.001)and even high tumor grade (≥G3; HR 2.84, 95% CI 1.17-31.36, *p* = 0.006) ,postoperative Chemotherapy(yes; HR 2.08, 95% CI 1.52-64.83, *p* = 0.032) and nodal metastasis at diagnosis (HR 11.7, 95% CI 4.96-49.91, *p* < 0.001) were associated with worse CSS. And the elevated fibrinogen value was also proved to be a prognostic factor of poor CSS according to the cut-off level. However, a fibrinogen cut-off of 340 mg/dl using ROC analysis was choose to be optimal in order to achieve higher prognostic accuracy (Figure 1). Accordingly, fibrinogen≥ 340 mg/dl with a hazard ratio (HR) of 6.97 (95% CI 1.84-22.97, *p* < 0.001) than the patient with fibrinogen < 340 mg/dl. The 5-year survival rate of all patients included (*n* = 72) was 62.4% for fibrinogen < 340 mg/dl(*n* = 41) and 41.9% for fibrinogen ≥340 mg/dl (*n* = 31, *p* = 0.023, log rank) (Figure 2).

**Figure 3:**
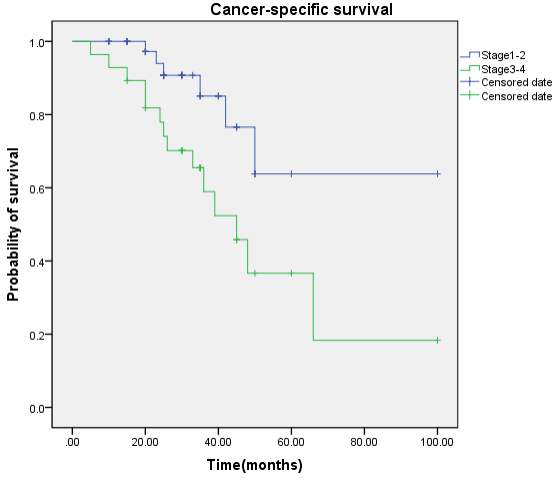
Cancer-specific survival (CSS) by tumor stage between the ≥ T2 and < T2 groups

**Figure 4:**
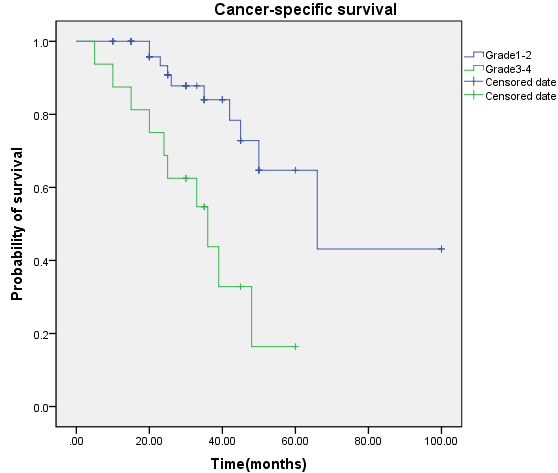
Cancer-specific survival (CSS) by tumor grade between the G1-2 and G3-4 groups

**Figure 5:**
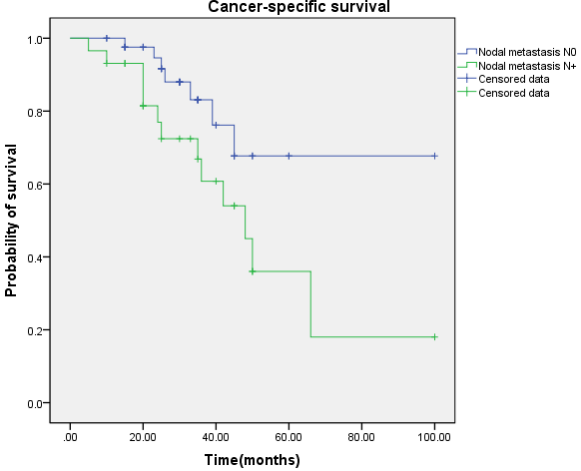
Cancer-specific survival (CSS) by nodal metastasis between the N0 and N+ groups

**Figure 6:**
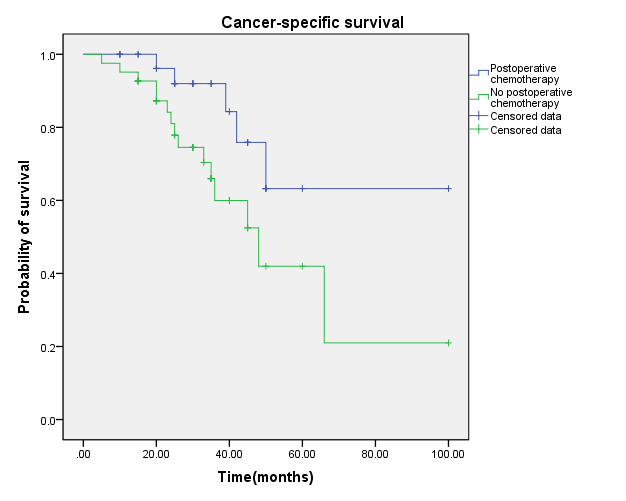
Cancer-specific survival (CSS) by postoperative chemotherapy between yes and no groups

**Figure 7:**
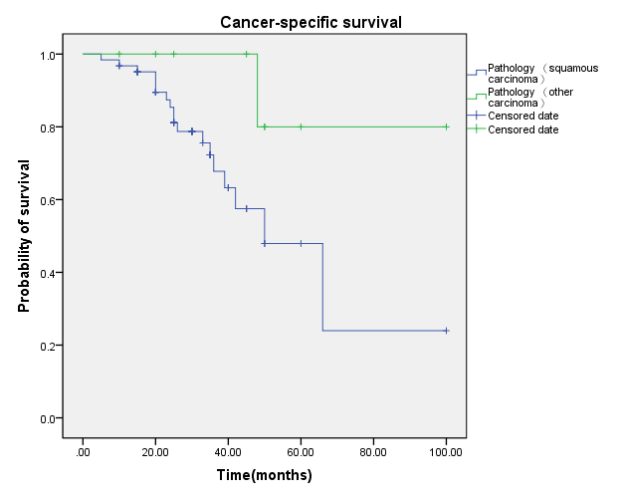
Cancer-specific survival (CSS) by pathological type between squamous carcinoma and other carcinoma groups

Multivariate regression analysis showed that the regional lymph node involvement or distant metastasis, tumor stage, tumor histological subtype, tumor grade, postoperative Chemotherapy were identified as significant and independent predict markers of CSS in penile cancer patient (Table 1, Figure 3, 4, 5, 6, 7). In contrast, smoking was not up to reach statistical significance. And these significance did not change when adopted step-wise backward LR-regression method analyses. However, focusing on the hyperfibrinogenemia of patients with fibrinogen≥340mg/dl, was defined as poor clinical outcome predictor in penile tumor patients.

## DISCUSSION

In the present research, it observed the clinical and prognostic significance between preoperative plasma fibrinogen levels and penile cancer. In the univariate analysis, smoke, tumor stage, tumor grade, postoperative chemotherapy, nodal metastasis, pathological type and peripheral plasma fibrinogen levels were associated with CSS. Multivariate survival analyses showed that nodal metastasis, tumor stage, pathological type, tumor grade, postoperative chemotherapy and preoperative plasma fibrinogen levels are significant independent prognostic markers for worse CSS in penile cancer patients As the traditional clinicopathological prognostic factors, such as diameter and number of the primary tumor, lymph node metastasis, perineural invasion, chemotherapy and pathological type have been generally used to predict survival for patient with malignant solid tumor. Vincenzo Ficarra et al. and Muneer, A. et al. reviewed the reported data concerning the prognostic markers in patients with penile squamous cell carcinoma (SCC) including tumor histologic grade, tumor histologic subtype, tumor pathologic extension and lymphatic and/or venous embolization, metastasis in regional lymph nodes, tumor growth pattern, p53, E-cadherin, MMP-2, and MMP-9 [5, 6]. Thereby, we summarize the molecular mechanisms about the connection between high levels of peripheral plasma fibrinogen and poor survival outcome of penile cancer patient.

Fibrinogen, as a major plasma protein, plays highly important roles in blood clotting, cellular and matrix interactions, and inflammation [7, 8]. Fibrinogen converted to insoluble fibrin by activated thrombin significantly affects blood clotting, fibrinolysis, inflammatory response, wound healing and neoplasia [9, 10]. Interleukin (IL)-6, immunosuppressive hormone, corticosteroid were reported to increase fibrinogen synthesis in the liver [11, 12] and IL-1 paradoxically suppresses fibrinogen production [13]. However, similar to inflammation, the mechanism of increased fibrinogen in tumor has not been clarified. Numerous published experimental research articles have demonstrated that fibrinogen plays a key role in cancer progression according to inducing tumor cell proliferation, epithelial-to-mesenchymal transition, migration, angiogenesis, hematogenous metastasis and postoperative recurrence [14–22]. Fibrinogen releases proliferative signals according to supplying as a scaffold for binding growth factors, such as FGF-2 [23]and VEGF [24]. Several growth factors binding could accelerate cellular adhesion, proliferation and migration during angiogenesis and cancer cell growth. Fibrinogen helps platelets to adhere to cancer cells, and platelets, in turn, induce more peripheral plasma fibrinogen to aggregate around cancer cells through forming thrombin. Several publications demonstrated the possibility that tumor cells themselves may generate fibrinogen [23, 25]. Thus, positive feedback cascade may blow up the activity of cancer cells and fibrinogen. While fibrinogen constantly present a role in tumor growth, invasion and metastasis through accelerating tumor neovascularization and according to accelerate the long-range adhesion of tumor cells [26, 27], the state of preoperative peripheral serum fibrinogen may reflect the progressive and metastatic possibility of tumors. On the other hand, fibrinogen and platelets participate in protecting cancer cells from natural killer cytotoxicity [28]. However, fibrinogen fragment is revealed to inhibit angiogenesis and consequently tumor progression by binding to and suppressing the function of integrins expressed on endothelial cells [18, 29, 30]. The effect of fibrinogen in cancer progression is also deemed to be disputed.

Lots of clinical articles have revealed that high preoperative peripheral plasma fibrinogen levels are associated with poor overall survival outcome and disease-free survival in various malignances. C. Perisanidis conducted a systematic review and meta-analysis to assess the prognostic effect of peripheral fibrinogen in solid tumors, showing that pretreatment plasma fibrinogen represents a biomarker of worse survival in tumor patients [31, 32]. However, the prognostic effect of preoperative peripheral serum fibrinogen level in penile cell carcinoma patients has not been elevated. Compared with all the results, high peripheral plasma fibrinogen levels were linked to disease CSS in penile cancer patients, and pretreatment peripheral plasma fibrinogen levels may be also an independent predictor for survival in operable penile cancer patients.

We were conscious of the shortages of a retrospective research that includes relatively less cases, and that the optimal cut off value of plasma fibrinogen was not defined. Prospective articles with a large population are warranted to define the accurate prognostic effect of pretherapeutic peripheral plasma fibrinogen levels in penile cancer patients. Moreover, systematic review of relative important others prognostic makers such as HPV or foreskin status, microscopic lymph vascular and perineural invasion, growth pattern were not included. Others, all potentially prognostic factors involved in the analysis were assessable in 72 patients, conducted in only one center with a small sample that it will not be generalized. All above potential limitations should be taken into account when we try to interpret the results.

## CONCLUSION

In conclusion, our research supply proof that the preoperative peripheral plasma fibrinogen level might be used as a new independent prognostic maker in penile cancer patients, which revealed that elevated peripheral plasma fibrinogen levels was associated with a worse CSS clinical result in patient. Compared to other experimental biomarkers, although our results await further validation, the plasma fibrinogen level is a cheaper and well defined laboratory factor, which makes our findings potentially applicable to clinical practice.
